# Gain-of-function defects in toll-like receptor 8 shed light on the interface between immune system and bone marrow failure disorders

**DOI:** 10.3389/fimmu.2022.935321

**Published:** 2022-09-02

**Authors:** Jack Bleesing

**Affiliations:** ^1^ Division of Bone Marrow Transplantation and Immune Deficiency, Cincinnati Children’s Hospital Medical Center, Cincinnati, OH, United States; ^2^ Department of Pediatrics, University of Cincinnati College of Medicine, Cincinnati, OH, United States

**Keywords:** inborn errors of immunity, bone marrow failure disorders, gain-of-function (GoF) mutation, infections, somatic mosacism

## Abstract

In this article, we will share lessons that patients with gain-of-function defects in Toll-like receptor 8 (TLR8-GOF) can teach us about the interface between bone marrow failure (BMF) disorders and inborn errors of immunity (IEI), subsequently referred to as “Interface Disorders”. TLR8-GOF is a relatively young entity (from a discovery standpoint) that—through both similar and dissimilar disease characteristics—can increase our understanding of interface disorders, for example, as it pertains to pathophysiology, the genetic mechanism of disease, and related diagnostics and therapeutics. From a genetics point of view, TLR8-GOF joins a growing list of (interface) disorders that can cause disease both with germline and somatic (mosaic) genetic variants. This not only has repercussions for the diagnostic workup of these disorders, inasmuch that routine genetic testing may miss somatic variants, but has therapeutic implications as well, for example, with the approach to curative treatment, such as hematopoietic stem cell transplantation. Following an introduction and schematic rendering of the interface, we will review the salient features of TLR8-GOF, with the understanding that the phenotype of this new disorder is likely not written in stone yet. In keeping with the principle of “Form Follows Function”, we will discuss specific immunological biomarkers that can be measured in clinical laboratories and highlight key disease features that pertain to TLR8-GOF, and can be found in several interface disorders. As can be seen from a schematic representation, the interface provides not only opportunities for learning and collaboration with respect to shared diagnostics but also the potential for drug repurposing and precision therapeutics. Ideally, collaboration also focuses on education and teaching, such that cross-fertilization and collaboration across these disciplines can create a framework for complementary research.

## An introduction to the interface

The traditional separation of cytopenias into mutually exclusive “destruction” versus “production” scenarios is no longer consistent with current knowledge, and, as such, one should not continue to assign these entities exclusively to the jurisdiction of a single subspeciality, such as hematology or immunology.

**Figure 1:**
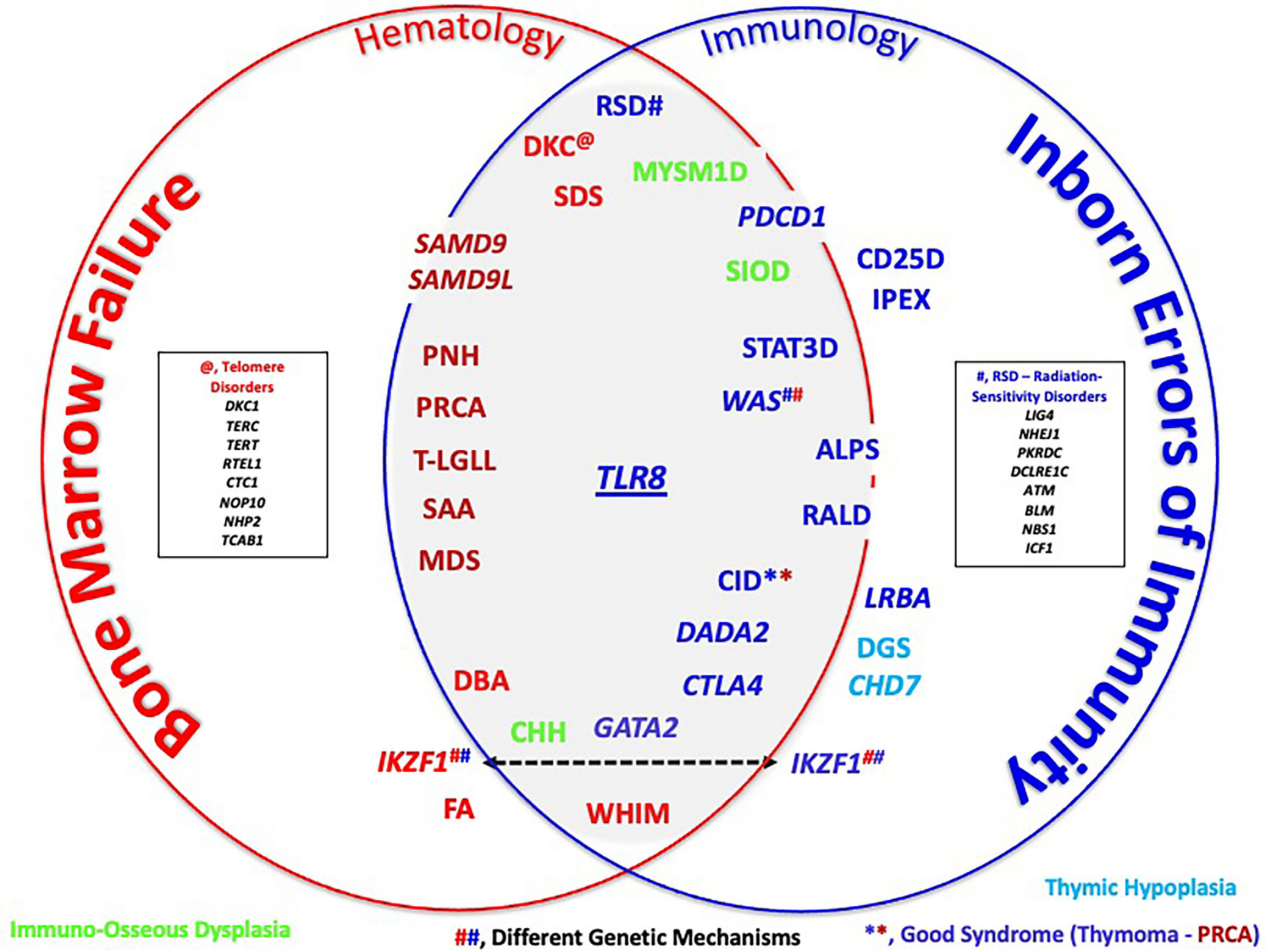
Schematic rendering of the bone marrow failure (BMF)/inborn errors of immunity (IEI) interface. Color codes arbitrarily refer to disorders that fit more with BMF (red) or more with IEI (dark blue). Light blue signifies the disorders of thymus development, while green represents immuno-osseous dysplasia disorders. Several genes, such as *IKZF1* and *WAS*, cause disease through different genetic mechanisms (e.g., loss-of-function mutations in WAS cause Wiskott–Aldrich syndrome, while gain-of-function mutations cause X-linked neutropenia). See **Table 1** for a detailed listing of disorders that one could find in or near the interface. Terms in italics refer to genes and non-italic to disorders. **, CID on the basis of Good Syndrome. ##, Different Genetic Mechanisms.

In the interface between these two (and perhaps more) subspecialties, one can discern a variety of interface disorders that although often (and traditionally) equated with either bone marrow failure (BMF) disorders or inborn errors of immunity (IEI), these disorders demonstrate variable (and dynamic) combinations of cytopenias due to errors in bone marrow function and defects in immune function (see a schematic of the interface and its gray zone in **Figure 1**).

This interface is a conceptual space and thus arbitrary in nature, with respect to content (what entities are “allowed entry” and where these entities are positioned in or near the interface). The goal is to make connections: between—seemingly—dissimilar conditions, between laboratory findings/observations, such as somatic rather than germline genotypes, usual cellular phenotypes, and connections between different subspecialties (e.g., hematology/oncology [H/O], allergy/immunology [A/I], and others).

The discovery of TLR8-GOF mutations will be used to frame this interface and show examples of approaches into the interface by entities that are more or less defined by bone marrow failure with concomitant autoimmune cytopenias (coming from the “H/O territory’) and entities with autoimmune cytopenias that also may be associated with bone marrow failure (coming from the A/I-rheumatology territory). Thus, the journey toward, and into, the interface conceptually starts in the clinic, where patients are first encountered and where we, as clinicians, are tasked with untangling often complex disorders.

To reiterate, the interface is not defined by molecular biological topography, as different entities arrive here through different pathogenic mechanisms. For example, while the disorders autoimmune lymphoproliferative syndrome (ALPS) (see the list of abbreviations) and LPS responsive beige-like anchor protein (LRBA) deficiency cause cytopenias due to autoantibody-mediated peripheral destruction, their molecular basis is different: ALPS is a disorder of defective apoptosis, while LRBA deficiency can be considered a so-called “Tregopathy” (disorder involving defective T regulatory T cells). Neither ALPS nor LRBA deficiency are known for bone marrow failure, but in the related Tregopathy, CTLA4-haploinsufficiency, cytopenias can be due to autoimmunity, as well as to bone marrow failure (1, 2). The deficiency of ADA2 (*DADA2*), a recent newcomer to the IEI-verse, also appears to mingle well with other interface disorders, consistent with the multisystem importance of the ADA2 enzyme (4–6). Certain interface disorders are associated with different disorders, depending on the genetic mechanism of disease. For example, WAS-LOF causes the IEI and Wiskott–Aldrich syndrome, while WAS-GOF causes X-linked congenital neutropenia (7).

Beyond ALPS, LRBA, CTLA4, ADA2, and WAS/XLN, the interface and its immediate surroundings are a busy place. In this schematic, disorders are (arbitrarily) grouped, based on their position within “BMF or IEI territories”, keeping in mind that the main purpose of the schematic is to illustrate the abundance of disorders that are in/near the interface and not “Subspecialty Ownership”. To make better sense of this interface, one can consider that a classification of these cytopenias would be based on distinct functional or pathobiological mechanisms. The examples of such classification would be disorders characterized by T-cell regulation defects (immune dysregulation, polyendocrinopathy, enteropathy, X-linked, CTLA4-haploinsufficiency), thymic hypoplasia (DGS, *CHD7*, *PAX1*), in which cytopenias are also due to defective T-cell development, regulation, as well as T-cell homeostasis, and immuno-osseous dysplasia (CHH, MYSM1, and SIOD), as well as telomere, ribosomal genesis and maturation disorders (“Ribosomopathies”), and radiation-sensitivity disorders, among others (8–14). In the H/O clinic, one might find patients with Fanconi anemia (FA), a rather classical BMF disorder. However, the immune system is not entirely normal such that autoimmunity can occur (and potentially make life more difficult in relation to stem cell transplantation) (15).

In summary, disorders in which T-cell and/or B-cell development, differentiation, function regulation, or homeostasis, including telomere maintenance and protection from DNA damage, are affected, either directly or indirectly (for example, through a chronic inflammatory environment), one should consider the two sides of the same coin of the destruction and production of blood and other cells and expect these disorders in the interface. Lastly, for a more detailed topography of the interface, the reader is referred to **Table 1**. This table is not intended to be all-inclusive but rather provide specific examples as these appear in the latest IUIS Classification (accessible through: https://link.springer.com/article/10.1007/s10875-022-01289-3, URL checked 25 July 2022) (3). As such, **Table 1** follows the IUIS Classification into Tables and Sections, while disorders that are not included in the IUIS Classification appear at the end (“REST”).

**Table 1:** Representative examples of Interface disorders (following the current IUIS Classification – see Reference 3).

2021 IUIS Table, Section	Subcategory	Disease	Gene[s]	Inheritance/Mechanism	OMIM
**Table 1, Section 2**	[S]CID*	RAG-def	*RAG1/RAG2*	AR	179615/179616
		DCLRE1C-def	*DCLRE1C*	AR	605988
		DNA PKcs-def	*PRKDC*	AR	615966
		Cernunnos/XLF-def	*NHEJ1*	AR	611290
		DNA ligase IV-def	*LIG4*	AR	601837
**Table 1, Section 3**	CID	CD3γ-def	*CD3G*	AR	186740
		ZAP70-def	*ZAP70*	AR	269840
		PAX1-def	*PAX1*	AR	615560
		HELIOS-def	*IKZF2*	AD/AR	NA
**Table 2, Section 2**	DNA Repair Defects	Ataxia-telangiectasia	*ATM*	AR	697585
		Nijmegen breakage S.	*NBS1*	AR	602667
		Bloom Syndrome	*BLM*	AR	210900
		ICF type 1	*DNMT3B*	AR	602900
		Hebo-def	*ERCC6L2*	AR	615667
**Table 2, Section 3**	Thymic Defects	DiGeorge Syndrome	*TBX1*	AD	602054
		CHARGE Syndrome	*CHD7*	AD	608892
**Table 2, Section 4**	Immuno-osseous Dysplasias	Cartilage Hair Hypoplasia	*RMRP*	AR	157660
		Schimke -IOD	*SMARCAL1*	AR	606622
		MYSM1-def	*MYSM1*	AR	612167
**Table 2, Section 5**	Hyper IgE Syndromes	PGM3-def	*PGM3*	AR	172100
**Table 2, Section 9**		PNP-def	*PNP*	AR	164050
		Kabuki S. (type 1/2)	*KMT2A/KDM6A*	AD/XLR	602113/300128
**Table 3, Section 2**		APDS	*PIK3CD*	AD-GOF	615513
		IKAROS-def	*IKZF1*	AD/haploinsuff.	603023
		PTEN-def	*PTEN*	AD	158350
		NFKB1-def	*NFKB1*	AD	164011
		TWEAK-def	*TNFSF12*	AD	602695
		TRNT1-def	*TRNT1*	AR	612907
		AID-def	*AICDA*	AR	605258
**Table 4, Section 3**	Tregopathy	IPEX	*FOXP3*	XLR	300292
		CD25-def	*IL2RA*	AR	147730
		CD122-def	*IL2RB*	AR	618495
		CTLA4-haploinsuff.	*CTLA4*	AD	123890
		LRBA-def	*LRBA*	AR	606453
		STAT3-GOF Disease	*STAT3*	AD-GOF	102582
		BACH2-def	*BACH2*	AD	605394
		IKAROS-GOF Disease	*IKZF1*	AD-GOF	NA
**Table 4, Section 4**		APECED (APS-1)	*AIRE*	AR or AD	240300
		TPP2-def	*TPP2*	AR	190470
		SOCS1-haploinsuff.	*SOCS1*	AD	619375
		PD1-def	*PDCD1*	AR	600244
**Table 4, Section 6**	ALPS	ALPS-FAS	*TNFRSF6*	AD OR AR	134637
		ALPS-FASLG	*TNFSF6*	AR	134638
		ALPS-CASP10	*CASP10*	AD	601762
**Table 5, Section 1**		X-linked Neutropenia	*WAS*	XL-GOF	300299
		Shwachman-Diamond S.	*SBDS, DNAJC21, EFL1*	AR	607444/617052/617941
**Table 5, Section 4**		GATA2-def	*GATA2*	AD	137295
**Table 6, Section 2**		WHIM	*CXCR4*	AD-GOF	162643
**Table 6, Section 6**		STAT1-GOF Disease	*STAT1*	AD-GOF	600555
**Table 6, Section 7**		TLR8-GOF Disease	*TLR8*	XL/XL-somatic	NA
**Table 9**	Fanconi Anemia (FA)	FA Type A-W (22 complementation groups)	*FANCA (+ 21 genes)*	AR (XLR)	227650 (see Table 9 for other 21)
	Telomeropathy	DKCX1	*DKC1*	XL	305000
		DKCA1, DKCA2, DKCA3, DKCA4, DKCA6	*TERC, TERT, TINF2, RTEL1, ACD*	AD	127550/187270/604319/616373/616553
		DKCB1-DKCB7	*NOLA3, NOLA2, WRAP53, TERT, RTEL1, PARN, ACD*	AR	224230/613987/613988/613989/615190/616353/616553
	Bone Marrow Failure S.	BMFS5	*TP53*	AD	618165
		MECOM-def	*MECOM*	AD	616738
		MIRAGE	*SAMD9*	AD-GOF	617053
		Ataxia-Pancytopenia S.	*SAMD9L*	AD-GOF	611170
**Table 10**	ALPS	ALPS-sFAS	*Somatic TNFRSF6*		
	RASopathy	RALD	*Somatic KRAS/NRAS - GOF*		
	*Unclassified*	VEXAS	*Somatic UBA1 (XL)*		
	CID	Good Syndrome (Thymoma)	unknown		
		TLR8-GOF Disease	*Somatic TLR8 (XL)*		
**REST**	Diamond-Blackfan Anemia (DBA, many types)	DBA-1	*DBA1*	AD (XLR)	105650
		Myelodysplastic Syndrome			NA
		PNH	*Somatic PIGA*		300818
		Pure Red Cell Aplasia (can be associated with thymoma)			NA
		Severe Aplastic Anemia (acquired condition)			NA

*, Usually manifesting as T-/B- SCID but can present as CID as well with certain genetic (hypomorphic) variants. NA, Not Applicable.

We will use the recently described disorder, defined by gain-of-function defects in Toll-like Receptor 8 (TLR8-GOF) as a model to explore the interface and connect similar and dissimilar disease characteristics among its constituents, hoping that this will increase our understanding of other interface disorders, for example, as it pertains to classification, pathophysiology, and related diagnostics and therapeutics.

## The new kid on the [interface] block

It is as if the interface was waiting for this newly described disorder, given that patients with TLR8-GOF defects appear to fit well among IEI and BMF disorders (16). Concentrated in its most essential ingredients, TLR8-GOF is a genetic disorder with autoimmune cytopenias, infections, lymphoproliferation, and neutropenia (16, 17). The mechanism of neutropenia, TLR8-GOF’s key feature, appears multifactorial with the aspects of both production and destruction mechanisms, based on antineutrophil antibodies, direct cytotoxicity and/or the Fas ligand–mediated destruction of neutrophils and progenitor cells, and impaired neutrophil differentiation and survival through pro-inflammatory mediators.

Most patients presented with infections, due to both common and uncommon microorganisms that appear to result from abnormal adaptive immunity and the consequences of prolonged neutropenia (e.g., *Aspergillus*). Several of the patients were considered to have ALPS, due to the evidence of lymphoproliferative disease (e.g., splenomegaly), cytopenias, and laboratory features, commonly seen in ALPS [e.g., double-negative T cells (DNTs), autoantibodies, and increased vitamin B12].

Although autoantibodies to blood cells were found, this was not a consistent finding, and one can conclude that neutropenia was not simply due to autoimmune destruction. Importantly, neutropenia, by and large, was unresponsive to standard-of-care immunosuppressive agents that are commonly used in interface disorders with autoimmunity, illustrating its multifactorial basis with features similar to BMF entities. Prolonged and/or unresponsive neutropenia was an indication for several of the patients to proceed to hematopoietic stem cell transplantation (HSCT).

The laboratory findings were diverse as well and—as commonly seen in the interface—dynamic over time. Several features stand out that include the presence of DNTs, CD4 T-cell lymphopenia, reduced isotype-switched memory B cells, associated with hypogammaglobulinemia, and increased pro-inflammatory cytokines. For a more detailed description of this complex and the new disorder, the reader is referred to (16, 17). Salient features of TLR8-GOF will be discussed in the following sections to connect to key aspects of other interface disorders, for example, with respect to the relevance of somatic mutations and the presence of large granular lymphocytes (LGL cells).

## Genetics/somatic mosaicism

There are two interesting features pertaining to the genetics of TLR8-GOF. The first concerns TLR8 itself. TLR8 is an endosomal receptor that senses microbial single-stranded RNA degradation products and serves to alert the immune system to the presence of viral and bacterial infections (17, 18). TLR8 is primarily expressed by neutrophils and monocytes. This is an interesting feature considering that many of the key features of TLR8-GOF pertain to the adaptive immune system (cellular and humoral immunity). This raises interesting questions regarding the pathogenesis of this disorder: how the increased function of a danger-signal receptor in innate leukocytes impacts on adaptive leukocytes, cumulating in a hyperinflammatory cytokine environment in the serum and bone marrow, linked to an activated T-cell phenotype and defects in B-cell maturation (16).

The second interesting feature of the TLR8-GOF story is the fact that all but one patient were found to have somatic mosaicism. Looking at the reported allele frequency, varying between 8%–26% of patients (**Table 1** in (16)), this is likely a reason why this disorder had eluded us until recently. Thus, the story of TLR8-GOF provides a good opportunity to review the topic of somatic mosaicism. Somatic mosaicism is increasingly recognized as a mechanism of disease that pertains to interface disorders as well (19, 20). Technical advances, including next-generation sequencing applications and digital droplet PCR platforms, have allowed the detection of somatic mosaicism in fewer cells, including the subsets of cells and at all stages of life.

Using TLR8-GOF as an example, we can ask several questions about somatic mosaicism (summarized in **Box 1**). The fact that most of the patients harbored (postzygotic) somatic variants (as opposed to the germline), reinforces the pathogenic impact of the gain-of-function mechanism of disease. This is also suggested by the fact that most IEI and related disorders with somatic mutations are due to a GOF mechanism, including those due to variants in KRAS, NRAS, NLRC4, NLRP3, NOD2, FAS, TMEM173, TNFRSF6, and TNFRSF1A) (19). Of note, five of these genes are linked to autoinflammatory disorders (Table 7 in the 2021 IUIS Classification). Also interesting is the fact that TLR8-GOF disease occurs—in full force—with only a minority of cells, typically leukocytes, affected by the somatic variant [variant allele frequency (VAF) between 8% and 26%]. In other somatic diseases, some of which can be found in our interface, the VAF appears to range from 1% to 50% (19, 20).

**BOX 1**

**Table:** 

Questions related to somatic mosaicism and interface disorders:
1] Which is more prominent: germline or somatic versions of the disease?
2] Are germline variants merely an extension of somatic variants or give somatic variants and germline variants rise to different entities (Noonan syndrome versus RALD)?
3] Is the mutational landscape the same, and/or are there somatic variants that have not been found in the germline setting (perhaps not compatible with life)?
4] What is the mechanism of the variant (e.g., LOF/GOF)?
5] What is the variant allele frequency (VAF), and how does it relate to phenotype?
6] Related—which cells/tissues are affected—contributing to the overall VAF, and what does that say about the origin of the somatic variant? Affected cells?
7] Is the VAF the same for affected cells/tissues? If not, what could be the reason (e.g., survival advantage), and is it stable over time?
8] What about contemporary next-generation sequencing (NGS) genetic testing and the detection of somatic variants**?**

In general, the impact of a somatic variant depends on the abundance (represented by the VAF), the identity of the variant (type of mutation), and the affected cell types, relative to the postzygotic origin. The TLR8-GOF patients nearly universally showed the variant in all nucleated blood cells, including lymphocytes (T-, B-, and Natural Killer (NK) cells), as well as monocytes (keeping in mind neutropenia in patients). In addition, the variant was found in cultured fibroblasts and/or saliva and (presumed) in lung tissue in one patient. By using digital droplet PCR, it appeared that the percentage of somatic versus wildtype alleles was relatively similar across those cells (but less than 30% in any cell/tissue). This appears to confirm that the origin of the mutation was in an early stage of embryonic development (16).

In other disorders, such as ALPS, RALD (Ras-associated lymphoproliferative disease), and NLRP3-associated “Inflammasomopathies”, mutations may occur at different stages along the pluripotent stem cell (PSC)–hematopoietic stem cell (HSC)–committed myeloid/lymphoid progenitor (CMP/CLP)–myeloid/lymphoid lineage (ML/LL) differentiation pathway (21–25). Variants in later stages, such as those committed to the lymphoid compartment, tend to arise later in life. In other words, PSC variants are present well before birth, while ML/LL variants originate, perhaps through a different driving force/mechanism, after birth and, in higher probability, with advancing age [e.g., T-cell LGL leukemia (T-LGLL)] (19). In fact, the acquisition of somatic variants, including in long-lived HSC (e.g., clonal hematopoiesis of indeterminate potential) is likely not that rare and may help explain not only the occurrence of certain blood cancers but also autoimmune diseases such as RA (with/without LGL cells) with advancing age (19).

As is succinctly noted in the paper by Van Horebeek et al. in (18), somatic variants are not aways the “Bad Guy”. Somatic reversion (or revertant mosaicism) refers to the spontaneous correction of a pathogenic mutation in a somatic cell. This process has been observed in many disorders, including IEI (for example, WAS, NEMO-IKBKG deficiency, ADA-SCID, and MYSM1) (26). Thus, somatic reversion has the potential of conveying a survival advantage to the involved cells/subset of cells, and thus may alleviate or modify the phenotype of the associated disorder, potentially moving it closer to or further from the interface. It will be interesting to see if/how the driving forces leading to somatic revertants are similar and dissimilar between different interface entities.

TLR8-GOF is likely not a new disease: it required more advanced technology to pick up the low variant allele frequency observed in the patients thus far. Standard variant calling methods are based on the presence of germline heterozygous mutations in approximately 50% of the sequence reads and may fail to detect somatic variants in allelic imbalance and lower frequencies and if there is insufficient coverage. Thus, allelic imbalance thresholds may need to be lowered to detect low-frequency variants, but this comes with the cost of substantially increasing candidate variants (19). The list of disorders with somatic variants is enriched with autoinflammatory disorders, such that this trade-off between lowering the VAF detection threshold and increasing variant identification should invite a careful evaluation of genetic testing results in patients with such disorders, as well as serve as a reminder that communications between a genetic testing entity and a physician should be bidirectional.

To rank potentially immune disease–relevant germline variants, gene-level strategies are used that are based on cumulative mutational damage to a particular human gene in the general population. This allows the removal of genes that are highly mutated in the general population and therefore less likely to cause severe disease. The 2% of genes in the genome with highest cumulative mutational damage (gene damage index) contribute a large proportion of the rare germline variants seen in the general population. Genes in which mosaicism is known to cause disease have a moderate-to-high intolerance to functional genetic variation. For example, NLRP3 is among the 10% of most intolerant genes. This has been used to explain why this gene has a high number of postzygotic variants among patients. For germline variants, pathogenicity is furthermore supported by variant-level strategies, for example, by the prediction of a variant to be damaging by a high combined annotation dependent depletion (CADD) score, adjusted by the mutation significance cutoff. The pathogenicity of somatic variants in monogenic immune disorders has similarly been supported by high CADD scores (19).

## Unusual suspects in the interface

**Figure 2:**
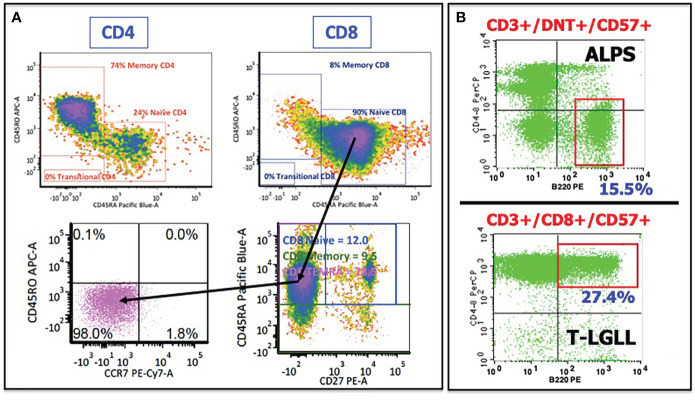
Illustrative flow cytometry cases showing abnormal T cells in several interface disorders. **(A)** Patient #3 from (8) demonstrated a discrepancy in CD45RO (memory) and CD45RA (naïve) expression between CD4+ T cells (upper left) and CD8+ T cells (upper right). Adding additional markers revealed that these CD45RA+/CD8+ T cells were not naïve T cells but rather TEMRAs (T-cell effector memory, RA-expressing), as determined by the lack of CD27 and CCR7 expression. **(B)** CD45RA-expressing T cells are also common in ALPS and in T-LGLL. Moreover, double-negative T cells in ALPS and T-LGL cells in T-LGLL express an unusual CD45RA isoform, B220. See text for further discussion.

In several patients, there was an abnormal presence of effector memory T cells expressing RA (TEMRAs), characterized by the expression of CD8 and CD45RA but lacking the other key markers of naïve T cells. These cells express the markers of cytotoxicity (CD57, granzymes, perforin), lack costimulatory markers (CD27, CD28), lack the homing/chemotaxis receptor CCR7, and are morphologically large. Traditionally, CD45RA, together with the expression of CCR7 and CD27/CD28, has been phenotypically equated with an antigen-naïve status of T cells. As an example, earlier phenotyping in TLR8-GOF patient #3 (in (16)) revealed—based on the costaining of CD45RA and CD45RO on CD4+ and CD8+ T cells, respectively—that there was a significant discrepancy between (presumed) naïve CD4+ and CD8+ T cells (see **Figure 2A**). Using the additional markers of naïve versus memory T cells, as described above, it became clear that these CD45RA+/CD8+ T cells were not naïve cells, but, in fact, TEMRAs, and corresponding to the large granular lymphocyte (LGL) cells found in his bone marrow on several occasions (27–31).

LGL cells were found in several patients, and are not unique to TLR8-GOF, given that the LGL cells of the T-cell origin feature prominently in the interface. In addition to TLR8-GOF, LGL cells can be found in several BMF entities, including severe aplastic anemia (SAA), paroxysmal nocturnal hemoglobinuria, and pure red cell aplasia (PRCA), as well as in autoimmune diseases (ALPS, RA/FS), hematopoietic malignancies, and plasma cell disorders (32–34). LGL cells may represent non-clonal reactive LGL cells, as well as indolent clonal proliferations (in patient #4 (16), this population was clonal in nature) and aggressive clonal neoplasms, such as T-LGLL and the chronic lymphoproliferative disorder of NK cells (CLPD-NK) (32, 33). Given the small numbers of TLR8-GOF patients thus far, it remains to be seen if LGL cells can undergo evolution from a reactive nature to a clonal process, and even to T-LGLL, but given the connection to TEMRAs, it raises interesting questions regarding the origin and relevance of TEMRAs in IEI and BMF. It also is incompletely understood whether LGL cells actively contribute to cellular destruction of blood cells (e.g., “anti-stem cell” or “anti-progenitor cell” autoimmunity) or are a consequence of the pro-inflammatory environment and/or other upstream consequences of abnormal TLR8 function in myeloid cells for the T-cell compartment. The TLR8 story would suggest the latter, keeping in mind that as far as we know so far, TLR8 is not expressed in the LGL cells themselves.

There are several other observations regarding TEMRAs/LGL cells in the interface worth mentioning or repeating. Firstly, abnormal T cells, whether DNT cells in ALPS, or LGL cells in T-LGLL, express an unusual CD45RA isoform, designated B220 (an example is shown in **Figure 2B**) (35). In addition to B220, these cells express TEMRA/LGL markers mentioned above (CD45RA, CD57) and lack CD27/CD28 and CCR7. The significance and relevance of B220 is not entirely clear, but it appears to be related to *in vivo* activation with defective homeostatic clearance through the Fas/Fas Ligand (FasL) pathway of apoptosis (36, 37). The Fas/FasL axis is defective in both ALPS and T-LGLL, albeit for different reasons (38). While this homeostasis pathway in patients with TLR8-GOF remains undetermined, Fas/FasL homeostasis invites further scrutiny, considering that several of the TLR8-GOF patients were considered to have ALPS at some point in their disease course and displayed DNT cells, elevated soluble vitamin B12, and FasL levels (16).

Secondly, somatic mutations feature prominently in the interface, whether in ALPS patients with somatic *FAS* mutations (largely confined to DNT cells), TLR8-GOF patients (multiple cell lineages consistent with a pluripotent stem cell origin), or patients with somatic STAT3 (and in certain cases, STAT5b) in LGL cells from T-LGLL patients (16, 32, 33, 39). This raises interesting questions regarding TEMRAs and LGL cells in the context of somatic mutations, as well as somatic revertants, and one should consider this an invitation to look at other interface disorders in a more detailed manner, for example, using digital droplet PCR methodology.

Lastly, in addition to LGL cells, the transcription factor STAT3 also has a rather interesting presence in the interface. As mentioned, somatic gain-of-function mutations in STAT3 occur in LGL cells in patients with T-LGLL, as well as in patients with LGL cells as part of PRCA. In addition, germline gain-of-function mutations in STAT3 underlie the IEI, STAT3 disease, which shares features with both ALPS and TLR8-GOF (40, 41). Lastly, abnormal STAT3 signaling has been observed in ALPS patients, in which it connects the pathognomonic elevated levels of IL-10 to sensitivity to BH3 mimetics (42). STAT3-associated sensitivity to BH3 mimetics has been observed in STAT3-GOF disease as well while in a potentially new addition to the interface; inherited PD-1 deficiency, a disorder characterized by mycobacterial infections, lymphoproliferation and autoimmunity, as well as abnormal STAT3 signaling, but without STAT3 mutations (43, 44).

Much remains to be learned about the usual and unusual suspects (cell types and biomarkers, etc.) in the interface. Shared and non-shared features may gain importance from a diagnostic standpoint as well; consider a uniform and extensive phenotyping of lymphocytes, the measurements of biomarkers and more sophisticated genomics (and other “-omics”) across interface disorders. In a sense, precision diagnostics might lead the way to precision therapeutics.

## Lessons for the transplant community

Congruent with a lack of understanding and the progressive nature of this new disorder, three TLR8-GOF patients of the cohort of six underwent allogeneic HSCT, which was successful in two, while the fourth patient perished before proceeding to HSCT (16). The main indications were prolonged and treatment-unresponsive neutropenia—reminiscent of the neutropenia seen in other interface disorders, such as SAA, as well as the presence of an LGL proliferation of a clonal nature in another patient. Although the numbers are small, one can appreciate that these were difficult transplants with significant pre-transplant HSCT complications.

What can the interface/transplant community learn from these initial cases? Summarized, genetic mosaicism, immunodeficiency, and neutropenia with serious infections, autoimmunity, proinflammatory cytokines in blood and marrow, dysregulated T cells with the potential of LGL development, and progression into a clonal T-LGL disorder point to potential icebergs on a perilous stem cell transplant journey.

Looking at these issues in more detail, the fact that full-blown TLR8-GOF was seen with a VAF below 20% in four patients suggests that nothing less than stable full myeloid engraftment is needed. Thus, the transplant approach should be regarded in the context of disorders in which “minimal residual disease” is unwanted. This will likely require (full) myeloablative conditioning regimens. Serotherapy and sufficient immunosuppressive therapy are likely warranted as well, appropriate to the donor source (match level), stem cell source (with/without T cells), and rejection potential (including T-cell-mediated rejection and through donor-specific antibodies). This can be achieved by antithymocyte globulin, alemtuzumab, and perhaps emapalumab, given the role of gamma-interferon in rejection (45). This, of course, needs to be balanced by (the potential) of severe viral infections and viral reactivation post-HSCT, particularly due to CMV, EBV, and adenovirus.

Full myeloablative regimens are less forgiving when it comes to transplant-related toxicity; a potential made worse by the preexistence of a proinflammatory environment and dysregulated/activated T- and B-cell compartments. Thus, consideration should be given to so-called “bridge therapy” (well) before the patient proceeds to HSCT. The constituent(s) of bridge-therapy regimes has/have not been worked out. One of several JAK inhibitors (so-called JAKinibs) have been suggested, and this approach might be of use in the preparation of HSCT in other interface disorders as well, for example, ADA2 deficiency and GOF-IEI affecting STAT1 and STAT3 that are associated with a pro-inflammatory diathesis and homeostatic derailment of T- and B-cell compartments (46, 47).

It is not in set in stone that patients with TLR8-GOF should proceed to HSCT. Thus, borrowing from other interface disorders, one could formulate HSCT indications based on clinical severity, including progression/treatment responses, as well as donor options, among others. For example, a pediatric patient with SAA might proceed to HSCT if a matched sibling is present but would start with immunosuppressive therapy if not (48).

Lastly, although TLR8-GOF is not T-LGLL, the above-described similarities between the two disorders (e.g., neutropenia, LGL cells, a pro-inflammatory environment, and somatic activating mutations) suggest that bridge therapy with JAK/STAT inhibitors (e.g., tofacitinib) may work in other interface disorders with prominent LGL cell proliferation, such as T-LGL as well (32).

## The communal space of the interface

Returning to the original description of the TLR8-GOF patients, one is reminded that detailed studies were completed to characterize the patients and their clinical, genetic, and immunological findings, with an attempt to answer the question why the gain of function of a microbial sensing gene in neutrophils and monocytes would lead to the rich tapestry of manifestations. Some of these studies can only be conducted in the research setting, but other parts of the discovery journey are routinely available to clinicians, working in the interface. As mentioned, a more detailed phenotyping and functional analysis of the immune system and characterization of the bone marrow, including the measuring and monitoring of the inflammatory environment, are more or less routinely used by both the IEI and BMF communities.

The fact that we are at the beginning of the TLR8-GOF learning curve, adding knowledge regarding the new kid on the block to the “Communal Knowledge-base” can focus attention on some commonalities, as described above. Questions then can be formulated regarding the interface as a whole, leading to a better understanding of existing entities and perhaps the identification of new disorders. One can envision that the conceptual Interface introduced here might be strengthened by a molecular/biological foundation, but this will work best if the IEI and BMF communities collaborate and learn from each other.

Finishing up, several goals for collaborative interactions come to mind:

- To define the interface with respect to its topography and boundaries, as well as identify a “common language” for the BMF and IEI fields, including defining indications and methods for the HSCT of interface disorders.
- To identity key stakeholders (“Guardians of the Interface”), barriers, and opportunities for collaborative projects.
- To use prototypic BMF and IEI disorders to further explore the interface and use as a framework to identify and characterize new interface disorders.
- To use our respective diagnostic tools and approaches to develop better (precision) diagnostics to screen and diagnose, stratify, and predict the prognosis and natural history of interface disorders, based on similar and dissimilar features.
- To move from disciplinary to transdisciplinary precision therapeutics and create a framework for trial design that may include drug repurposing.
- To develop an agenda for transdisciplinary education and training (of the next generation).

## Author contributions

The author confirms being the sole contributor of this work and has approved it for publication.

## Conflict of interest

The authors declares that the research was conducted in the absence of any commercial or financial relationships that could be construed as a potential conflict of interest.

## Publisher’s note

All claims expressed in this article are solely those of the authors and do not necessarily represent those of their affiliated organizations, or those of the publisher, the editors and the reviewers. Any product that may be evaluated in this article, or claim that may be made by its manufacturer, is not guaranteed or endorsed by the publisher.

## Glossary

**Table:**

ALPS	Autoimmune lymphoproliferative syndrome
CD25D	CD25 deficiency
CHH	Cartilage hair hypoplasia
CID	Combined immunodeficiency
CHD7	Chromodomainhelicase-DNA-binding protein 7
CTLA4	Cytotoxic Tlymphocyte-associated protein 4
DADA2	Deficiency of the enzyme ADA2 (adenosine deaminase 2)
DBA	Diamond blackfan anemia
DGS	DiGeorge syndrome
DKC	Dyskeratosis congenita
DNT	Double-negative T cells
FA	Fanconi anemia
GATA2	GATA binding protein 2
HSCT	Hematopoietic stem cell transplantation
IKZF1	IKAROS family zinc finger 1
IPEX	Immune dysregulation, polyendocrinopathy, enteropathy, X-linked
LGL	Large granular lymphocyte
LRBA	LPS responsive beige-like anchor protein
MDS	Myelodysplastic syndrome
MYSM1	Myb-like
SWIRM	and MPN domains 1
PNH	Paroxysmal nocturnal hemoglobinuria
PRCA	Pure red cell aplasia
PDCD1	Programmed cell death protein 1
RA/FS	Rheumatoid arthritis/Felty syndrome
RALD	Ras-associated autoimmune leukoproliferative disorder
RSD	Radiation-sensitivity disorders
SAA	Severe aplastic anemia
SAMD9[L]	Sterile alpha motif domain containing 9 [like]
DS	Shwachman– Diamond syndrome
SIOD	Schimke immuno-osseous dysplasia
STAT3-D	Signal transducer and activator of transcription 3-disease
TEMRA	T-cell effector memory, RAexpressing
T-LGLL	T-large granular lymphocyte leukemia;
TLR8	Toll-like receptor 8 [gain-of-function]
VAF	Variant allele frequency
WAS	Wiskott–Aldrich syndrome
WHIM	Warts, hypogammaglobulinemia, infections, myelokathexis
